# Induction of Tertiary Phase Epileptiform Discharges after Postasphyxial Infusion of a Toll-Like Receptor 7 Agonist in Preterm Fetal Sheep

**DOI:** 10.3390/ijms22126593

**Published:** 2021-06-19

**Authors:** Kenta H.T. Cho, Mhoyra Fraser, Bing Xu, Justin M. Dean, Alistair J. Gunn, Laura Bennet

**Affiliations:** 1 The Department of Physiology, The University of Auckland, Auckland 1023, New Zealand; kenta.cho@auckland.ac.nz (K.H.T.C.); m.fraser@auckland.ac.nz (M.F.); j.dean@auckland.ac.nz (J.M.D.); l.bennet@auckland.ac.nz (L.B.); 2 Shenzhen Bay Laboratory, Shenzhen 518118, China; xubing@szbl.ac.cn

**Keywords:** cardiovascular, electrophysiology, fetal sheep, hypoxia–ischemia, Toll-like receptors

## Abstract

Background: Toll-like receptor (TLR) agonists are key immunomodulatory factors that can markedly ameliorate or exacerbate hypoxic–ischemic brain injury. We recently demonstrated that central infusion of the TLR7 agonist Gardiquimod (GDQ) following asphyxia was highly neuroprotective after 3 days but not 7 days of recovery. We hypothesize that this apparent transient neuroprotection is associated with modulation of seizure-genic processes and hemodynamic control. Methods: Fetuses received sham asphyxia or asphyxia induced by umbilical cord occlusion (20.9 ± 0.5 min) and were monitored continuously for 7 days. GDQ 3.34 mg or vehicle were infused intracerebroventricularly from 1 to 4 h after asphyxia. Results: GDQ infusion was associated with sustained moderate hypertension that resolved after 72 h recovery. Electrophysiologically, GDQ infusion was associated with reduced number and burden of postasphyxial seizures in the first 18 h of recovery (*p* < 0.05). Subsequently, GDQ was associated with induction of slow rhythmic epileptiform discharges (EDs) from 72 to 96 h of recovery (*p* < 0.05 vs asphyxia + vehicle). The total burden of EDs was associated with reduced numbers of neurons in the caudate nucleus (*r*^2^ = 0.61, *p* < 0.05) and CA1/2 hippocampal region (*r*^2^ = 0.66, *p* < 0.05). Conclusion: These data demonstrate that TLR7 activation by GDQ modulated blood pressure and suppressed seizures in the early phase of postasphyxial recovery, with subsequent prolonged induction of epileptiform activity. Speculatively, this may reflect delayed loss of early protection or contribute to differential neuronal survival in subcortical regions.

## 1. Introduction

Hypoxic–ischemic encephalopathy (HIE) is more frequent in preterm infants than at term [1] and is highly associated with increased risk of adverse outcomes, such as subcortical brain injury and life-long neurodevelopmental disabilities [2,3]. There is increasing evidence that hypoxia–ischemia (HI) activates secondary inflammatory pathways and that modulation of these pathways has potential to improve outcomes [4,5,6,7,8,9].

Toll-like receptors (TLRs) are key regulators of innate immunity, and although they are often considered to have damaging roles within the developing brain, they can also reduce injury largely via preconditioning (tolerance) [10,11,12]. For example, we previously reported that central infusion of the synthetic TLR7 agonist Gardiquimod (GDQ) from 1 to 4 h after asphyxia in preterm fetal sheep reduced white and grey matter damage after 3 days recovery [13]. Interestingly, in this cohort, central GDQ administration was associated with hypertension and transiently improved recovery of spectral edge frequency (SEF), and suppression of epileptiform transients [14]. In a subsequent study using the same protocol to assess whether neuroprotection was persistent, we found no significant improvement in total or immature/mature oligodendrocytes in the periventricular and intragyral white matter tracts after 7 days recovery [15]. Intriguingly, in that study, GDQ was also associated with increased neuronal survival in the CA4 region of the hippocampus but greater neuronal loss in the caudate nucleus.

In the present study, we examined the hypothesis that this apparent transient histological neuroprotection after an early TLR7 agonist infusion after asphyxia, in preterm fetal sheep at 0.7 gestation [15], is associated with modulation of seizure-genic processes and hemodynamic control. Neural development at this fetal age is broadly equivalent to 27–30 weeks in the human infant [16].

## 2. Results

### 2.1. Umbilical Cord Occlusion and Fetal Biochemistry

There was no significant difference in the duration of umbilical cord occlusion (UCO) between asphyxia + vehicle and asphyxia + GDQ (21.4 ± 0.5 vs. 20.4 ± 0.5 min, respectively, *p* > 0.05). There were no significant differences in pH, blood gases, glucose and lactate between groups in the baseline period, during UCO or for the first few days after UCO (Table 1). The asphyxia + GDQ group showed transient changes in pH and PaCO_2_ values between 120 and 144 h post-UCO (*p* < 0.05 vs. sham asphyxia) and a small reduction in lactate between 72 and 120 h post-UCO (*p* < 0.05 vs. asphyxia + vehicle).

### 2.2. Blood Pressure

**Figure 1:**
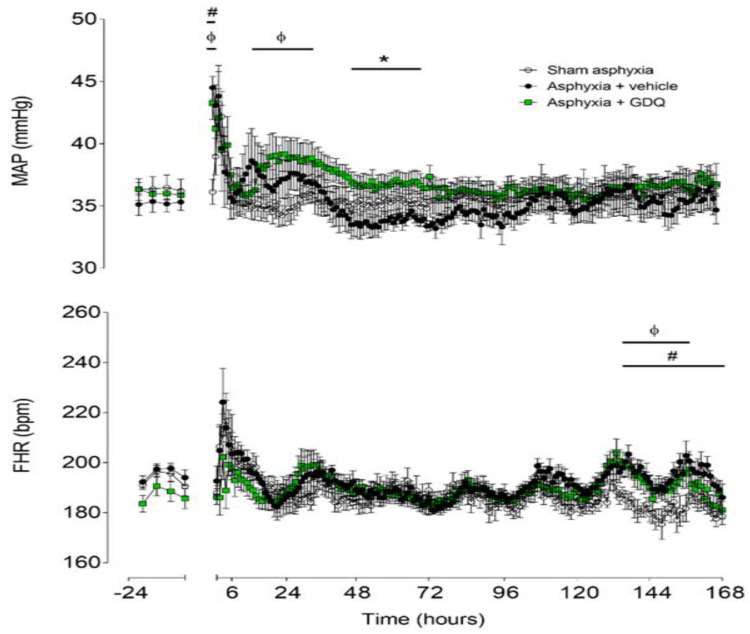
Time sequence of changes in fetal mean arterial pressure (MAP) (**top**) and fetal heart rate (FHR) (**bottom**) from 24 h before until 168 h after umbilical cord occlusion (UCO) in sham asphyxia (open circles, n = 8; 4 female, 4 male), asphyxia + vehicle (black circles, n = 7; 4 female, 3 male) and asphyxia + Gardiquimod (GDQ) (green squares, n = 7; 4 female, 3 male) animals. The period of UCO is not shown. Data are mean ± SEM; 6 h averages before UCO and 1 h averages after UCO. Statistical significance was determined by repeated measures ANOVA followed by Fisher’s LSD post hoc test: * *p* < 0.05, asphyxia + GDQ vs. asphyxia + vehicle; ^ф^ *p* < 0.05, sham asphyxia vs. asphyxia + GDQ; ^#^ *p* < 0.05, sham asphyxia vs. asphyxia + vehicle.

Baseline mean arterial blood pressure (MAP) was not significantly different between the groups (Figure 1). UCO (data not shown) was associated with similar profound hypotension in the asphyxia groups, compared to sham asphyxia (last minute of UCO: sham asphyxia 36.7 ± 1.1 vs. asphyxia + vehicle 12.6 ± 0.8 and asphyxia + GDQ 11.7 ± 0.8 mmHg; *p* < 0.05). After UCO, there was a transient increase in MAP in the asphyxia + vehicle group for 2 h (*p* < 0.05 vs. sham asphyxia), followed by resolution to baseline values. The asphyxia + GDQ group showed a similar increase in MAP for 2 h (*p* < 0.05 vs. sham asphyxia), followed by a significant increase from 16 to 36 h after UCO (*p* < 0.05, vs. sham asphyxia) and from 48 to 72 h post-UCO (*p* < 0.05, vs. asphyxia + vehicle). There was no significant correlation between peak MAP after asphyxia and NeuN-positive cells in the caudate (*r*^2^ = 0.018, *p* > 0.05) or putamen (*r*^2^ = 0.021, *p* > 0.05).

### 2.3. Fetal Heart Rate

Baseline fetal heart rate (FHR) was not significantly different between groups (Figure 1). UCO, but not sham asphyxia, was associated with rapid onset of bradycardia (last minute of UCO: sham asphyxia 182.9 ± 6.1 vs. asphyxia + vehicle 68.7 ± 5.0 and asphyxia + GDQ 68.7 ± 3.4 bpm; *p* < 0.05). FHR recovered rapidly after release of UCO. The asphyxia + vehicle group showed increased FHR from 133 to 168 h after UCO (*p* < 0.05 vs. sham asphyxia). Similarly, the asphyxia + GDQ group showed increased FHR from 133 to 156 h after UCO (*p* < 0.05 vs. sham asphyxia) but was not significantly different from the asphyxia + vehicle group.

### 2.4. Electroencephalogram and Spectral Edge Frequency

**Figure 2:**
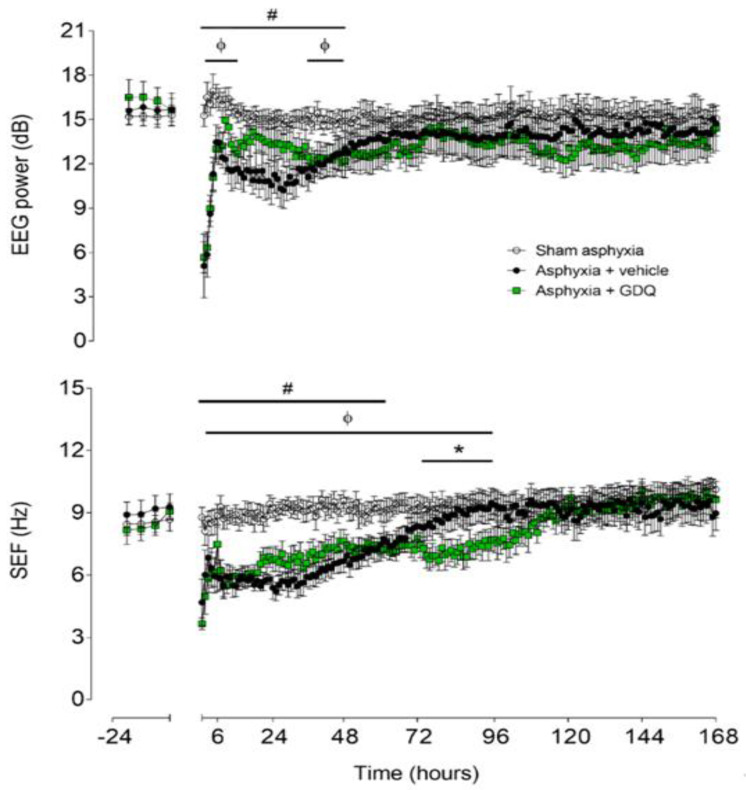
Time sequence of changes in fetal electroencephalographic (EEG) power (**top**) and spectral edge frequency (SEF) (**bottom**) from 24 h before until 168 h after umbilical cord occlusion (UCO) in the sham asphyxia (open circles, n = 7; 4 female, 3 male), asphyxia + vehicle (black circles, n = 6; 3 female, 3 male) and asphyxia + GDQ (green squares, n = 7; 4 female, 3 male) animals. The period of UCO is not shown. Data are mean ± SEM; 6 h averages before UCO and 1 h averages after UCO. Statistical significance was determined by repeated measures ANOVA followed by Fisher’s LSD post hoc analysis: * *p* < 0.05, asphyxia + GDQ vs. asphyxia + vehicle; ^ф^ *p* < 0.05, sham asphyxia vs. asphyxia + GDQ; ^#^ *p* < 0.05, sham asphyxia vs. asphyxia + vehicle.

**Figure 3:**
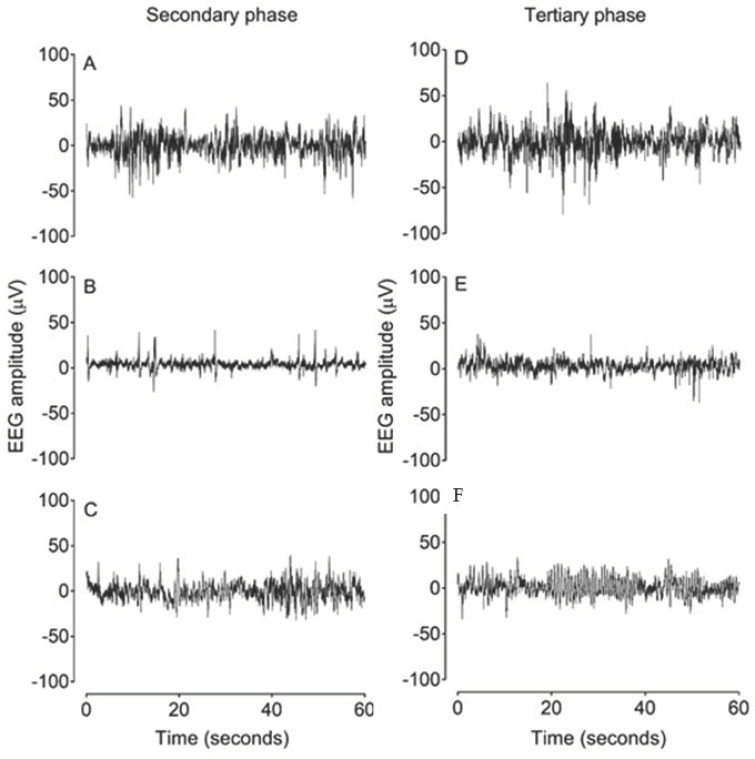
Examples of raw EEG activity from sham asphyxia, asphyxia + vehicle and asphyxia + GDQ groups in the secondary phase at 18 h after umbilical cord occlusion (UCO) (**A**–**C**) and in the tertiary phase at 84 h after UCO (**D**–**F**). Panels (**A**) and (**D**) demonstrate normal discontinuous mixed amplitude and frequency observed in preterm fetal sheep at 0.7 gestation. Panel (**B**) demonstrates suppressed background activity interspersed with the presence of sharp and fast wave transients in the asphyxia + vehicle group in the secondary phase. Panel (**C**) shows greater background activity of higher amplitude in the asphyxia + GDQ group. Panel (**E**) demonstrates a mildly suppressed background activity in the asphyxia + vehicle group in the tertiary phase. Panel (**C**) shows that the reduction in SEF seen in the asphyxia + GDQ group in Figure 2 is associated with slow rhythmic epileptiform discharge (ED) activity.

There were no significant differences in electroencephalographic (EEG) power or SEF between groups in the baseline period (Figure 2). In the asphyxia + vehicle group, EEG power remained significantly suppressed until 48 h post-UCO (*p* < 0.05 vs. sham asphyxia). In the asphyxia + GDQ group, EEG power was significantly suppressed between 2 and 15 h and between 36 and 48 h post-UCO (*p* < 0.05 vs. sham asphyxia), with a transient recovery in EEG power between 16 and 36 h post-UCO, which was not significantly different to the asphyxia + vehicle group. This transient increase in EEG power in the asphyxia + GDQ group corresponded with a greater background activity on raw EEG records (Figure 3). In the asphyxia + vehicle group, SEF was significantly suppressed until 60 h post-UCO (*p* < 0.05 vs. sham asphyxia). In the asphyxia + GDQ group, SEF remained suppressed until 96 h post-UCO (*p* < 0.05 vs. sham asphyxia) and was not different from the asphyxia + vehicle group except for a significant reduction at 72–96 h (*p* < 0.05 vs. asphyxia + vehicle).

### 2.5. Postasphyxial Seizure Activity

**Figure 4:**
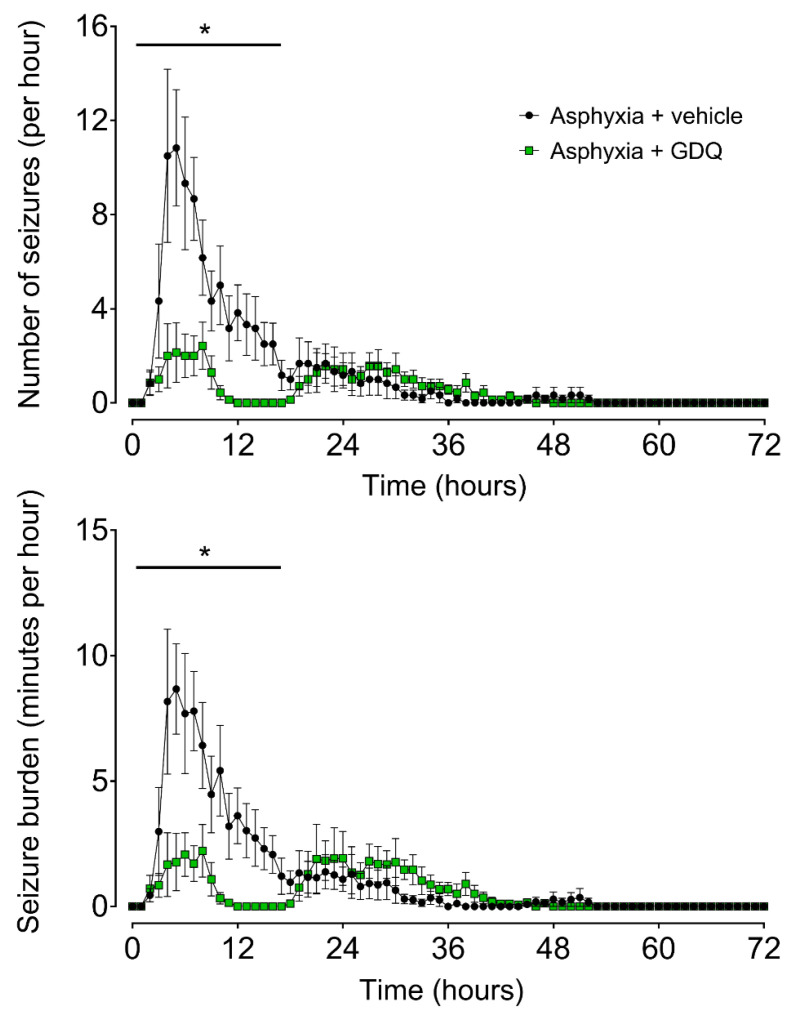
Time sequence of fetal seizure activity after umbilical cord occlusion (UCO). The numbers of seizures per hour (**top**) and the total seizure burden per hour (**bottom**) in asphyxia + vehicle (black circles, n = 6; 3 female, 3 male) and asphyxia + GDQ (green squares, n = 7; 4 female, 3 male). Data are mean ± SEM. Statistical significance was determined by repeated measures ANOVA followed by Fisher’s LSD post hoc analysis: * *p* < 0.05, asphyxia + GDQ vs. asphyxia + vehicle.

Delayed onset of stereotypic evolving seizures occurred in both asphyxia groups. The mean seizure number and seizure burden per hour were significantly lower in the asphyxia + GDQ group during the first 18 h post-UCO (*p* < 0.05 vs. asphyxia + vehicle; Figure 4). Individual seizures were significantly longer in duration in the asphyxia + GDQ group (*p* < 0.05 vs. asphyxia + vehicle; Table 2), although there were no significant differences in onset of seizures, resolution of seizures, total seizure duration or peak amplitude.

### 2.6. Slow-Wave EEG Activity

**Figure 5:**
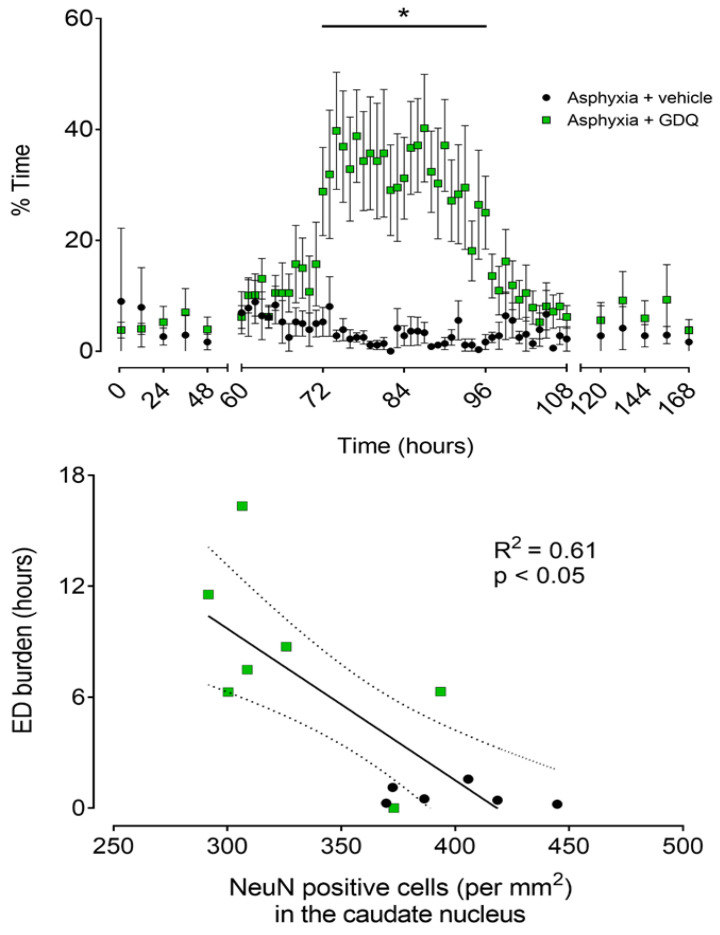
Top panel: Time sequence of changes in percentage time per hour of epileptiform discharge (ED) activity after umbilical cord occlusion (UCO) in asphyxia + vehicle (black circles, n = 6; 3 female, 3 male) and asphyxia + GDQ (green squares, n =7; 4 female, 3 male) groups. Data between 0 and 48 h and between 120 and 168 h post-UCO were batch analyzed at 12 h intervals. Data between 60 and 108 h post-UCO are 1 h averages. Data are mean ± SEM. Statistical significance was determined by repeated measures ANOVA followed by Fisher’s LSD post hoc analysis: * *p* < 0.05, asphyxia + GDQ vs. asphyxia + vehicle. Bottom panel: Relationship between the sum of all minutes of epileptiform discharge (ED burden, hours), measured between 72 and 96 h post-UCO, and the number of surviving neurons (NeuN-positive cells/mm^2^) in the striatal caudate nucleus after 7 days recovery for the asphyxia groups (asphyxia + vehicle and asphyxia + GDQ, *r*^2^ = 0.61, *p* < 0.05, black solid line). Dashed lines are the 95% confidence intervals. Green squares: asphyxia + GDQ; black circles: asphyxia + vehicle.

The reduction in SEF between 72 and 96 h post-UCO in the asphyxia + GDQ group corresponded with the appearance of slow rhythmic epileptiform discharge (ED) activity on continuous EEG records (Figure 3). These waveforms occurred in a much greater proportion of the EEG recordings in the asphyxia + GDQ group from 72 to 96 h post-UCO (*p* < 0.05 vs. asphyxia + vehicle; Figure 5). Linear regression showed that the sum of all minutes of ED activity (ED burden) between 72 and 96 h post-UCO was negatively correlated with survival of NeuN-positive neurons in the caudate nucleus (*r*^2^ = 0.61, *p* < 0.05; Figure 5) and the CA1/2 hippocampal region (*r*^2^ = 0.66, *p* < 0.05; not shown). By contrast, there was no significant correlation between ED burden and survival of NeuN-positive neurons in the putamen, CA3 and CA4 hippocampal regions, dentate gyrus, thalamic medial nucleus and the thalamic medial geniculate nucleus (*p* > 0.05, not shown).

## 3. Discussion

It is now well established that neural injury evolves after acute HI, offering a potential window for treatment. In the early recovery or latent phase, mitochondrial activity and oxidative metabolism typically recover partially or completely for up to 6 h, whereas EEG activity remains highly suppressed [17]. This is followed by delayed deterioration over approximately 72 h in a secondary phase, as shown by delayed onset of seizures, loss of mitochondrial activity and neuronal loss. Seizures typically resolve by 48–72 h. However, there is increasing evidence that cell loss may continue to evolve over a long period of time, in a tertiary phase of injury [18]. There is considerable evidence that most interventions are most effective if they are started as early as possible in the latent phase [19], but this may not be sufficient for long-lasting neuroprotection.

The present study demonstrates that infusion of GDQ from 1 to 4 h significantly reduced the number and burden of seizures up to 18 h after UCO. Subsequently, however, GDQ was associated with very delayed development of slow rhythmic ED activity in the early tertiary phase of recovery (from 72 to 96 h). Greater numbers of EDs were strongly associated with more severe injury, as shown by reduced neuronal survival in the caudate nucleus and CA1/2 hippocampal region, whereas no association was found in the putamen, CA3 and CA4 hippocampal regions, dentate gyrus or the thalamic medial and geniculate nuclei. Finally, GDQ was associated with sustained postasphyxial hypertension, similar to our previous report [14], that resolved after 72 h. These findings highlight the potential for TLR agonists, such as GDQ, to modulate seizures following an asphyxia insult, but to induce delayed epileptiform activity, which may contribute to differential susceptibility of specific subcortical regions to neuronal injury.

Seizures are common after perinatal HI. Although they are highly associated with adverse outcomes [20,21], there is controversy as to whether seizures substantially modulate HI brain injury, and thus should be treated [22,23,24,25]. Similarly to previous reports in this preterm sheep model [17], in the present study, there was delayed onset of seizures in the asphyxia + vehicle group after 6 h, which largely resolved by 18 h of recovery. While little is known about the temporal evolution of preterm seizures in humans, these data are consistent with the finding in normothermic term infants that seizures are maximal in the first 24 h after HI [26,27].

GDQ administration was associated with transiently improved recovery of background EEG activity, similarly to our previous report [14], and reduced seizures in the secondary phase. These data are consistent with growing evidence that immunomodulators are associated with functional improvement and reduced seizures in animal models of HIE [28,29]. The specific mechanism of seizure suppression is unclear. However, there is growing evidence that cytokines can modulate neuronal excitability [30,31,32]. Supporting this hypothesis, the peak fetal plasma concentrations of interleukin (IL)-10 and tumor necrosis factor (TNF)-α in the present cohort [15] and in our previous study using the same model [14] correspond with the greatest suppression of seizures in the asphyxia + GDQ group. Both these cytokines are known to exert anticonvulsant effects. For example, the threshold temperature for hyperthermia-induced seizures in juvenile rats treated intranasally with IL-10 is increased compared with saline-treated controls [33].

In fetal sheep, antagonists of N-methyl-D-aspartic acid (NMDA)-receptor activation potently suppress postischemic seizures [34] and IL-10 can inhibit NMDA-receptor-mediated intracellular calcium signaling in rat hippocampal neurons [35] and reduce excitotoxic brain lesions in P5 mice [36]. Similarly, TNF-α protected hippocampal neurons against glutamate-receptor-induced neurotoxicity [37] and significantly reduced kainic acid induced seizures via activation of TNF receptor 2 [38]. However, TNF-α can also exert proconvulsive effects [39] in a concentration-dependent manner [38,40].

Intriguingly, in the present study, GDQ was associated with sustained reduction of SEF between 72 and 96 h after asphyxia. This reduced EEG frequency was related to a dramatic increase in numbers of slow rhythmic EDs compared with the asphyxia + vehicle group. The mechanism is unknown. At least potentially, this could reflect a direct or indirect epileptogenic effect of TLR7 activation and downstream inflammatory processes [30]. There is evidence, for example, that acute administration of TLR3 and TLR4 agonists in the neonatal rat can increase expression of excitatory NMDA and AMPA glutamate receptor subunits for weeks after treatment, with an associated increase in susceptibility to epileptiform activity and inflammation-dependent seizures [41,42]. There could also be a role for chronic inflammation via nuclear factor kappa light chain enhancer of activated B cells (NF-κB)-dependent transcription of inflammatory molecules, which modulate the expression of genes involved in neurogenesis, cell death and synaptic reorganization and plasticity—processes that occur concomitantly with epileptogenesis [31,43]. Further investigations of these mechanisms are required.

Alternatively, this delayed epileptiform activity may simply reflect the very delayed evolution of neuronal loss after early treatment with GDQ, and in particular loss of inhibitory interneurons [44]. Interestingly, there was greater neuronal loss in the caudate nucleus after GDQ infusion compared with vehicle [15], with a significant negative linear relationship between ED burden and numbers of surviving neurons in the caudate nucleus. This raises the possibility that EDs may contribute to loss of overall neuroprotection in some regions, and even exaggerate injury in the caudate nucleus. In support, there is consistent evidence in the preterm fetal sheep linking epileptiform transient activity, including spikes, sharps and slow waves, during the latent phase of recovery after asphyxia with greater neuronal loss in the caudate nucleus [23,45]. Equally, in human cases of metabolic encephalopathy and cerebral HI, EDs are associated with loss of inhibitory GABA-ergic interneurons, which may lead to disinhibition and propagation of epileptiform events [44]. Further research is essential to determine whether anticonvulsant therapy would improve neuronal survival.

Consistent with our previous report [14], infusion of GDQ led to a mild, sustained hypertension, which resolved 72 h after asphyxia. This increase in fetal blood pressure was not attributable to increased FHR, supporting that it was mediated by increased peripheral vascular resistance. Similarly, female mice treated with imiquimod, a TLR7 agonist, had significantly increased MAP after 8 weeks, without changes in heart rate, and exhibited vascular remodeling in the mesenteric arteries and reduced endothelium-dependent vasodilator responses to acetylcholine in the aorta [46]. These cardiovascular alterations were associated with reduced nitric oxide availability, increased oxidative stress and greater mRNA expression of proinflammatory cytokines, including interferon (IFN)-α, IFN-γ, IL-1β, IL-6 and IL-17. In preterm newborns, elevated blood pressure may increase the risk of intraventricular hemorrhage [47]. Reassuringly, and consistent with evidence that long-term prognosis is unaffected for infants with hypertension that resolves over time [48,49], in the present study, GDQ-mediated fetal hypertension resolved after 3 days recovery and was not associated with neuronal survival, and no hemorrhagic complications were observed.

Some potential limitations of this study should be considered. We have not undertaken GDQ dose–response studies, although we have demonstrated substantial histological neuroprotection after 3 days recovery with the same regime [13]. Furthermore, we did not examine the effects of GDQ on the normal brain. This will be important for future studies, to address issues such as whether changes in seizures and ED reflect treatment alone or modulation of the seizure threshold and/or other neuroexcitatory processes following HI [50,51].

In conclusion, the present study demonstrates that acute intraventricular administration of the TLR7 agonist GDQ following asphyxia was associated with suppression of overt seizures in the early secondary phase after profound asphyxia but was followed by delayed induction of ED and loss of early neuroprotection after 7 days recovery. This pattern suggests that late ED activity may either reflect or even contribute to substantial tertiary phase neuronal loss within the caudate nucleus after 7 days postasphyxial recovery, leading to loss of early neuroprotection. This finding raises the important question of whether either a more prolonged infusion of GDQ or a repeated regimen might offer durable neuroprotection. Finally, our current data demonstrate that as part of the development of neuroprotective therapies for HIE, including immunomodulatory therapies, it is important to examine the long-term effects on EEG parameters to assess the impact of potentially pathologic epileptiform events on neural outcome.

## 4. Materials and Methods

### 4.1. Ethics

All procedures undertaken in this study were approved by the Animal Ethics Committee of the University of Auckland and were performed in accordance with the New Zealand Animal Welfare Act 1999 and the University of Auckland’s Code of Ethical Conduct for the use of animals for teaching and research. This study is compliant with the ARRIVE guidelines for reported animal research [52]. The pH, blood gas, postmortem body and brain weight and histopathological outcomes have been reported [15]. For clarity, pH and blood gas parameters at selected time points are reported in Table 1.

### 4.2. Animals and Surgical Preparations

Twenty-two Romney–Suffolk cross fetal sheep were instrumented at 98–99 days of gestation (term is ~147 days gestation) as previously reported in detail [15]. Ewes were given an intramuscular injection of oxytetracycline (20 mg/kg, Phoenix Pharm, Auckland, New Zealand) before surgery and anesthetized by intravenous propofol (5 mg/kg; AstraZeneca Limited, Auckland, New Zealand). Anesthesia was maintained with 2–3% isoflurane in O_2_ (Bomac Animal Health, Hornsby, NSW, Australia). Ewes were given a continuous infusion of saline (250 mL/h) through an intravenous line to maintain fluid balance. Animals were monitored throughout surgery by trained anesthetic technicians.

The uterus was exposed through a midline incision and the fetus was partially exteriorized for instrumentation. Polyvinyl catheters were placed into the right and left brachial arteries to measure fetal blood pressure and for preductal blood sampling. To measure amniotic fluid pressure, a catheter was placed in the amniotic sac. To record the fetal electrocardiogram (ECG), electrodes (AS633-3SSF; Cooner Wire Company, Chatsworth, CA, USA) were placed in the subcutaneous space over the right shoulder and at the level of the left fifth intercostal space. To measure fetal EEG activity, two pairs of electrodes (AS633-7SSF; Cooner Wire Company) were placed on the dura over the parasagittal parietal cortex bilaterally (10 and 15 mm anterior, 5 mm lateral to bregma), with a reference electrode over the occiput. To infuse GDQ, an ICV catheter was placed in the left lateral cerebral ventricle (6 mm anterior and 4 mm lateral to bregma) to a depth of 1.0 cm. ICV administration was chosen to provide initial proof of concept of the neural effects of GDQ after asphyxia, without confounding by changes in the blood–brain barrier. Finally, an inflatable silicone occluder (OC16HD; 16 mm, In Vivo Metric, Healdsburg, CA, USA) was placed loosely around the umbilical cord.

Before the uterus was closed, ~500 mL warm sterile saline to replace amniotic fluid and 80 mg gentamicin (Pfizer, Auckland, New Zealand) were added to the amniotic sac. The maternal midline skin incision was infiltrated with a local analgesic (10 mL 0.5% bupivacaine plus adrenaline; AstraZeneca Limited). All electrode leads and polyvinyl catheters were exteriorized via the maternal flank and the maternal saphenous vein was catheterized for postoperative maternal care and euthanasia.

### 4.3. Postoperative Care

After surgery, animals were housed in temperature-controlled rooms (16 ± 1 °C, humidity 50 ± 10%) with a 12 h light/dark cycle, in individual metabolic cages. Ewes were provided with water and food ad libitum (pelleted grass; Dunstan Nutrition, Hamilton, New Zealand). Animals recovered for 4–5 days before starting experiments. Antibiotics were given daily to the ewe for 3 days (600 mg benzylpenicillin sodium, Novartis, Auckland, New Zealand, and 80 mg gentamicin, Pfizer). Fetal and maternal vascular catheters were maintained patent by continuous infusion of heparinized saline (20 U/mL at a rate of 0.15–0.20 mL/h). Daily fetal arterial blood samples were collected to measure preductal pH, blood gas and base excess (ABL800 Flex analyzer, Radiometer, Auckland, New Zealand) and glucose and lactate (YSI model 2300, Yellow Springs, OH, USA) to assess fetal health.

### 4.4. Physiological Monitoring

All physiological data were recorded continuously from 24 h before UCO until 7 days after UCO by computer using custom data acquisition software (LabVIEW for Windows; National Instruments, Austin, TX, USA). Data included fetal MAP (Novatrans II transducers, MX860; Medex, Hilliard, OH, USA), FHR derived from the ECG signal and EEG. MAP was corrected for maternal lying and standing by subtracting amniotic fluid pressure.

The MAP signal was filtered with an analog fifth-order low-pass Butterworth filter with a cut-off frequency at 20 Hz, then digitized at a sampling rate of 512 Hz. The raw ECG signal was filtered with an analog first-order high-pass filter with a cut-off frequency of 0.05 Hz and an analog fifth-order low-pass Bessel filter with a cut-off at 100 Hz. The ECG was then digitized at a sampling rate of 1024 Hz. RR intervals were extracted from this signal to calculate fetal heart rate. EEG signals were amplified 10,000x and processed with an analog first-order high-pass filter with a cut-off at 1.6 Hz and an analog fifth-order low-pass Butterworth filter with a cut-off at 500 Hz and digitized at 4096 Hz. The EEG signal was then filtered by a low-pass filter with a digital IIR Type 2 Chebyshev filter with a cut-off frequency of 120 Hz and decimated to 256 Hz for analysis of EEG waveforms for seizures. Total EEG power (µV2) was calculated from the power spectrum between 1 and 20 Hz and log transformed for presentation (dB, 10 x log10 (power)). SEF was calculated as the frequency below which 90% of EEG power was present, within the 1–20 Hz band.

### 4.5. Experimental Protocol

At 103–104 days of gestation, animals were randomly assigned to sham asphyxia (n = 8), asphyxia + vehicle (n = 7) or asphyxia + GDQ (n = 7) groups. Fetuses of either sex were included in the study (sham asphyxia = 4 female, 4 male; asphyxia + vehicle = 4 female, 3 male; asphyxia + GDQ = 4 female, 3 male). Fetal asphyxia was induced at 7:30 a.m. by rapid, complete inflation of the umbilical cord occluder for up to 24 min or until blood pressure fell below 8 mmHg or there was fetal asystole lasting more than 20 s [53]. Sham asphyxia animals received no occlusion. This protocol is associated with moderate subcortical neuronal loss [15], comparable to that observed in preterm infants [54].

ICV infusions were performed using a CMA-100 microinjection pump (Carnegie Medicin, Torshamnsgatan, Sweden). Asphyxia + GDQ fetuses received a primed continuous infusion of 3.34 mg of GDQ (InvivoGen, San Diego, CA, USA), approximately equivalent to 1.8 mg/kg of fetal body weight, dissolved in 2 mL of sterile endotoxin-free modified artificial cerebrospinal fluid (aCSF) [55], at a rate of 11.1 μL/min from 1 to 4 h after the end of occlusion. We have previously shown that this dose reduces white and grey matter injury after 3 days recovery in this fetal sheep model [13]. The sham asphyxia and asphyxia + vehicle animals received an infusion of the vehicle alone (aCSF) using the same infusion protocol. Fetal arterial blood pH, blood gases and glucose and lactate levels were measured at 1 h before (baseline), during (5 and 17 min) and after UCO (4, 6, 24, 48 and 72 h). Ewes and fetuses were killed seven days after UCO, by overdose of sodium pentobarbital given i.v. to the ewe (9 g Pentobarb 300, Chemstock International, Christchurch, New Zealand). At postmortem, fetal body and brain weights were measured. Placement of the ICV catheter was verified at postmortem and then on histological examination.

### 4.6. Data Acquisition and Statistical Analysis

Analysis of all physiological data was performed using a custom analysis program (LabVIEW for Windows; National Instruments). The baseline period for the analysis of all physiological data was taken as the mean of the 24 h period before occlusion. All physiological data following asphyxia were assessed as hourly averages.

Each minute of the raw EEG recording was analyzed manually, by a single assessor blinded to groups, for the presence of seizures using a program allowing visualization of EEG records at a resolution of 2 s. Seizures were defined as the concurrent appearance of sudden, repetitive and rhythmic waveforms in the EEG signal lasting >10 s with a stereotypic evolving nature [56] and a minimum seizure amplitude of 20 μV. This definition ensured that seizures were easily distinguished from background activity [56]. Numbers of high-amplitude seizures per hour, duration of seizures per hour (seizure burden per hour), total number of seizures, sum of all minutes of seizures (total seizure burden), the individual duration of seizures, peak amplitude, time of seizure onset and resolution and the total duration of seizures (from first to last seizure) were evaluated. A seizure was assigned to a specific minute if the seizure started in that minute.

Continuous raw EEG traces in both asphyxia groups were analyzed each minute between 60 and 108 h post-UCO and batch analyzed at 12 h intervals between 1 and 60 and between 108 and 168 h post-UCO to determine the percentage of time showing repetitive slow rhythmic ED activity. These waveforms were defined by a period of 200–350 milliseconds from trough to peak, with waveforms forming consistent events lasting >10 s (Figure 3) [57,58,59]. Signal artifacts prevented EEG and seizure analysis in one fetus in each of the sham asphyxia and asphyxia + vehicle groups.

Statistical analysis was performed using SPSS v25 (IBM, Armonk, NY, USA). The changes over time of cardiovascular and electrophysiological parameters were evaluated by analysis of variance (ANOVA) with time treated as a repeated measure. The baseline, infusion and recovery periods were evaluated separately. Post hoc comparisons were made using Fisher’s protected least significant difference (LSD) when a significant effect of group or an interaction between group and time was found. The effect of infusion was analyzed from 1 to 4 h after UCO. Fetal biochemical parameters were evaluated by one-way ANOVA followed by Fisher’s protected LSD post hoc test.

We measured the mean duration and maximum amplitude of individual seizures, the time of onset and resolution of seizures, the corresponding total duration of seizures and the total seizure number and burden. Groups were compared by one-way ANOVA. Postasphyxial changes in ED activity and the number and burden of seizures per hour were analyzed by ANOVA with time as a repeated measure followed by Fisher’s protected LSD post hoc test when a significant overall effect was found. Linear regression analysis was performed to compare the relationship between the sum of all minutes of EDs (ED burden), between 72 and 96 h post-UCO, and the survival of neurons (NeuN-positive neurons) at 7 days postasphyxial recovery (as reported [15]). Statistical significance was accepted when *p* < 0.05. Data are presented as mean ± standard error of the mean [17].

## Figures and Tables

**Table 1:** Fetal blood gases, pH and metabolites during the baseline period, asphyxia and recovery.

Group	Baseline	17 min UCO	+1 h	+4 h	+24 h	+72 h	+96 h	+120 h	+144 h	+168 h
*pH*										
Sham asphyxia	7.36 ± 0.01	7.36 ± 0.00	7.37 ± 0.00	7.37 ± 0.01	7.35 ± 0.00	7.35 ± 0.01	7.35 ± 0.00	7.34 ± 0.01	7.35 ± 0.01	7.35 ± 0.01
Asphyxia + vehicle	7.36 ± 0.01	6.81 ± 0.01 *	7.30 ± 0.00 *	7.36 ± 0.02	7.37 ± 0.01	7.36 ± 0.01	7.36 ± 0.01	7.36 ± 0.01	7.35 ± 0.01	7.36 ± 0.01
Asphyxia + GDQ	7.38 ± 0.01	6.82 ± 0.01 *	7.32 ± 0.01 *	7.39 ± 0.01	7.37 ± 0.01	7.37 ± 0.01	7.37 ± 0.01	7.37 ± 0.01 *	7.37 ± 0.00 *	7.37 ± 0.01
*P_a_CO_2_ (mmHg)*										
Sham asphyxia	52.4 ± 1.2	51.2 ± 1.5	54.3 ± 1.3	52.7 ± 1.4	55.8 ± 1.3	54.4 ± 1.3	51.9 ± 1.6	54.7 ± 1.4	55.3 ± 2.2	53.0 ± 1.8
Asphyxia + vehicle	51.4 ± 1.1	150.7 ± 2.5 *	48.9 ± 1.2 *	51.9 ± 1.1	50.1 ± 1.9 *	50.3 ± 1.6	50.5 ± 1.6	52.3 ± 1.6	51.6 ± 1.7	51.3 ± 2.2
Asphyxia + GDQ	50.9 ± 1.2	147.0 ± 2.3 *	48.6 ± 1.3 *	49.6 ± 0.7	49.2 ± 1.5 *	47.5 ± 1.5 *	47.9 ± 1.4	48.6 ± 1.0 *	49.7 ± 0.6 *	49.2 ± 1.8
*P_a_O_2_ (mmHg)*										
Sham asphyxia	28.9 ± 1.4	27.7 ± 1.2	29.0 ± 1.1	28.6 ± 1.7	28.8 ± 1.8	29.6 ± 1.3	28.3 ± 2.1	28.4 ± 0.7	27.6 ± 1.0	28.9 ± 0.9
Asphyxia + vehicle	26.8 ± 0.7	5.8 ± 0.4 *	31.8 ± 2.6	25.7 ± 1.6	29.9 ± 2.0	31.8 ± 1.8	30.7 ± 2.2	28.6 ± 1.8	29.1 ± 1.8	28.7 ± 2.1
Asphyxia + GDQ	28.3 ± 1.1	5.7 ± 0.4 *	31.9 ± 1.3	27.3 ± 1.4	30.9 ± 1.4	35.0 ± 1.8	33.6 ± 1.4	31.2 ± 1.3	31.0 ± 1.2	29.5 ± 1.3
*Lactate (mmol/L)*										
Sham asphyxia	0.8 ± 0.0	0.7 ± 0.0	0.8 ± 0.0	2.5 ± 0.5	0.8 ± 0.0	0.8 ± 0.1	0.8 ± 0.1	0.8 ± 0.1	0.8 ± 0.1	0.9 ± 0.1
Asphyxia + vehicle	0.9 ± 0.1	6.8 ± 0.1 *	4.4 ± 0.2 *	3.8 ± 0.5	1.4 ± 0.2 *	1.0 ± 0.1	0.9 ± 0.1	0.9 ± 0.1	0.9 ± 0.1	0.9 ± 0.1
Asphyxia + GDQ	0.8 ± 0.0	6.5 ± 0.3 *	4.0 ± 0.1 *	3.0 ± 0.6	1.1 ± 0.1	0.7 ± 0.0 ^#^	0.7 ± 0.0 ^#^	0.7 ± 0.0 ^#^	0.7 ± 0.0	0.7 ± 0.0
*Glucose (mmol/L)*										
Sham asphyxia	1.3 ± 0.1	1.3 ± 0.1	1.4 ± 0.1	1.6 ± 0.1	1.2 ± 0.0	1.4 ± 0.1	1.3 ± 0.1	1.3 ± 0.1	1.3 ± 0.1	1.4 ± 0.1
Asphyxia + vehicle	1.3 ± 0.1	0.7 ± 0.1 *	1.7 ± 0.1 *	1.5 ± 0.1	1.5 ± 0.1	1.4 ± 0.1	1.4 ± 0.1	1.2 ± 0.1	1.2 ± 0.1	1.3 ± 0.0
Asphyxia + GDQ	1.3 ± 0.1	0.7 ± 0.2 *	1.7 ± 0.1 *	1.6 ± 0.1	1.5 ± 0.2	1.5 ± 0.1	1.4 ± 0.1	1.3 ± 0.1	1.3 ± 0.1	1.4 ± 0.1

Data are presented as mean ± SEM. Abbreviations: GDQ, Gardiquimod; UCO, umbilical cord occlusion; PaCO_2_, arterial pressure of carbon dioxide; PaO_2_, arterial pressure of oxygen. Statistical significance was determined by one-way ANOVA, followed by Fisher’s least significant difference post hoc analysis. * *p* < 0.05 vs. sham asphyxia; ^#^ *p* < 0.05 vs. asphyxia + vehicle.

**Table 2:** Characteristics of seizures after umbilical cord occlusion.

Group	Seizure Onset (h)	Seizure End (h)	Total Seizure Period (h)	Total Number of Seizures	Total Seizure Burden (h)	Max. Amplitude (µV)	Individual Seizure Duration (s)	Average Burden (min/h)
Asphyxia + vehicle	3.6 ± 0.5	29.2 ± 6.4	25.6 ± 6.1	99.2 ± 24.8	12.1 ± 3.2	101.6 ± 16.2	53.7 ± 1.0	4.1 ± 0.6
Asphyxia + GDQ	4.6 ± 0.9	35.8 ± 5.3	31.5 ± 4.9	37.6 ± 9.4 *	6.9 ± 1.8 *	116.2 ± 13.4	65.6 ± 3.9*	2.2 ± 0.4 *

Data are mean ± SEM. Abbreviations: GDQ, Gardiquimod. Statistical significance was determined by 1-way ANOVA: * *p* < 0.05, asphyxia + GDQ vs. asphyxia + vehicle.

## Data Availability

Data are available from the corresponding author on reasonable request.
